# Understanding Farmers' Behavior and Their Decision-Making Process in the Context of Cattle Diseases: A Review of Theories and Approaches

**DOI:** 10.3389/fvets.2021.687699

**Published:** 2021-12-02

**Authors:** Marit M. Biesheuvel, Inge M. G. A. Santman-Berends, Herman W. Barkema, Caroline Ritter, John Berezowski, Maria Guelbenzu, Jasmeet Kaler

**Affiliations:** ^1^Department of Production Animal Health, Faculty of Veterinary Medicine, University of Calgary, Calgary, AB, Canada; ^2^Research and Development Epidemiology, Royal GD, Deventer, Netherlands; ^3^Department of Health Management, Atlantic Veterinary College, University of Prince Edward Island, Charlottetown, PE, Canada; ^4^Schotland's Rural College, Inverness, United Kingdom; ^5^Veterinary Sciences Division, Agri Food and Biosciences Institute, Belfast, Ireland; ^6^School of Veterinary Medicine and Science, University of Nottingham, Nottingham, United Kingdom

**Keywords:** infectious disease, farmers, veterinarians, cattle, behavioral determinants

## Abstract

Understanding farmers' behavior regarding disease control is essential to successfully implement behavior change interventions that improve uptake of best practices. A literature review was conducted to identify theoretical underpinnings, analytical methodologies, and key behavioral determinants that have been described to understand farmers' behavior in disease control and prevention on cattle farms. Overall, 166 peer-reviewed manuscripts from studies conducted in 27 countries were identified. In the past decade, there were increasing reports on farmers' motivators and barriers, but no indication of application of appropriate social science methods. Furthermore, the majority (58%) of reviewed studies lacked a theoretical framework in their study design. However, when a theoretical underpinning was applied, the Theory of Planned Behavior was most commonly used (14% of total). The complexity of factors impacting farmers' behavior was illustrated when mapping all described key constructs of the reviewed papers in behavior change frameworks, such as the socioecological framework and the Capability, Opportunity and Motivation Behavior (COM-B) model. Constructs related to personal influences and relationships between farmers and veterinarians were overrepresented, whereas constructs related to other interpersonal and contextual environments were not extensively studied. There was a general lack of use of validated scales to measure constructs and empirically validated theoretical frameworks to understand and predict farmers' behavior. Furthermore, studies mainly focused on measurements of intention of stakeholder behavior rather than actual behavior, although the former is a poor predictor of the latter. Finally, there is still a lack of robust evidence of behavior change interventions or techniques that result in a successful change in farmers' behavior. We concluded that for a sustainable behavior change, studies should include wider constructs at individual, interpersonal, and contextual levels. Furthermore, the use of empirically validated constructs and theoretical frameworks is encouraged. By using coherent frameworks, researchers could link constructs to design interventions, and thereby take the first step toward theory-driven, evidence-based interventions to influence farmers' behavior for disease control.

## Introduction

Although evidence-based practices are available to prevent and control most diseases affecting domestic farm animals, economic losses due to animal health issues remain substantial (1). Researchers emphasize the importance of risk factors related to numerous cattle diseases, but on-farm understanding and implementation of disease prevention and control measures is often suboptimal [e.g., (2–4)]. For example, only 27% of producers in north-west England described biosecurity in relation to the management of pathogens or diseases on farms. Although farmers reported biosecurity measurements to be useful, many farmers did not actually apply them (2). Additionally, despite comprehensive extension efforts and low costs to producers, 35% of Alberta's dairy farms were not enrolled in the voluntary Johne's disease control program initiated in 2012 (5). It was previously assumed that decision-making processes were mainly driven by aspects related to financial costs and benefits. However, there is increasing evidence that decisions are also influenced by a variety of additional factors, e.g., farmers' perceived risk, perceived knowledge, perceived control, incentivization, emotions, and normative beliefs (6–10). Therefore, better understanding of farmers' processes of decision-making regarding disease control on cattle farms is necessary to understand suboptimal implementation of best practices. Also, to design and implement successful behavioral change intervention studies, better understanding of farmers' behavioral influences is crucial.

Failure to implement evidence-based risk mitigating practices was also noticed regarding human health measures (11, 12). Disciplines such as sociology, anthropology, health psychology, and economics provide insights into factors that impact behavior. Social science approaches in veterinary epidemiology are increasingly recognized to understand the impact of stakeholders' behavior on disease control. Nevertheless, knowledge is still lacking on theoretically underpinned key determinants impacting whether farmers adopt practices that are important to prevent and control cattle diseases (13). Explicit use of theory can help to identify influences on behavior change, understand mechanisms of change, and inform implementation of interventions (14). However, interventions are often developed without a systematic method and without reference to evidence or to theories produced by behavioral or social sciences (12).

Also, current veterinary epidemiological infectious disease transmission models often fail to incorporate behavior constructs and simply assume homogeneity in farmers' behavior (13). However, human behavior is influenced by a variety of factors and individual farmers are likely to respond differently in their evaluation of risk and whether to apply a disease control measure, with important consequences for disease transmission. The recent COVID-19 pandemic highlighted that social aspects are important drivers for local and global variations in disease dynamics and burdens (15). Therefore, a better understanding of important constructs that influence farmers' behavior would facilitate incorporation of human factors in epidemiological disease models, and eventually improve predictions and results.

The aims of this review were to: (1) explore the use of psychosocial theory, sociological approaches, and analytical methodologies in research studying farmers' behavior in the context of cattle disease control; and (2) map the identified key constructs into comprehensive behavioral frameworks.

## Measuring Farmers' Behavior

A strong theoretical framework should reveal existing predispositions about a study and can assist in data coding and interpretation (16). Generally, a theory is an organization of many ideas with a high degree of explanatory power that provides guidance regarding methods that will answer the research question. Moreover, theories explain difficult social interactions and phenomena and enable the explanatory process to become more explicit (16).

Psychology and rural sociology with various theoretical lenses are two common and widely used disciplines that have contributed toward understanding behavior in health allied fields and agriculture, especially farmers' behavior. Although other disciplines, e.g., anthropology and economics, have also contributed to the field, those are beyond the scope of this review. Below we describe psychosocial theories and rural sociology concepts.

### Psychosocial Theories and Approaches

Theory in the context of human behavior indicates why, when and how a behavior does or does not occur (12). Furthermore, theories identify determinants of influence to be targeted to alter behavior and they reflect integrated knowledge about relevant mechanisms of action and moderators of change of behavior.

Davis et al. (17) identified 82 health psychology theories for describing human behavior. Every theory consists of various key constructs, i.e., specialized terms, to label the theory's elements (18). The ‘Theory of Planned Behavior’ (TPB) (19), an extension of the ‘Theory of Reasoned Action’ (20) is the most commonly used psychosocial theory in human health (21). A central factor in TPB is an individual's intention to perform a given behavior, because it is assumed that intention captures motivational factors that influence a behavior (19). The TPB consists of three conceptually independent determinants of intention. The first is ‘attitude toward the behavior,’ referring to the degree to which a person has a favorable or unfavorable appraisal of the behavior in question (19). Secondly, ‘subjective norms’ refer to perceived social pressures toward a certain behavior, and thirdly, ‘perceived behavioral control’ refers to the perceived ease or difficulty of performing a behavior. The latter includes a reflection on past experiences as well as anticipated impediments and obstacles (19).

According to a systematic review (22), the other most common theories used in human health behavior include the Transtheoretical Model (23), the Social Cognitive theory (24), and the Health Belief Model (25). The Transtheoretical Model conceptualizes behavior change as a process involving a series of six distinct stages: pre-contemplation, contemplation, preparation, action, maintenance, and termination. The Social Cognitive theory (24) states that behavior is influenced directly by goals and self-efficacy expectations and indirectly by self-efficacy, outcome expectations, and sociostructural factors. The Health Belief Model (25) hypothesizes that health-related behavior depends on a combination of perceived susceptibility, perceived severity, perceived benefits, perceived barriers, cues to action, and self-efficacy. These theories include overlapping constructs such as results expectancies, i.e., beliefs about the behavior and expectations for the results of the behavior (21). Additionally, overlap in self-evaluation occurs, described as the individual's subjective evaluation of the amount of control and competency to engage successfully in a behavior. Finally, there is an overlap in social factors, regarded as the influences of other people on behavior change (21).

It is noteworthy that these health psychology theories are mainly driven by an individuals' cognitive processes and actions and individual-level factors (as above) relating to motivation and capability (17, 26). Limited attention has been given to ‘external influences’ and wider interpersonal factors that shape outcomes and behaviors.

In contrast to psychological theories, sociological approaches focus predominantly on the context in which people live and interact. They consider behavior as an outcome of complex inter-relationships and shared social practice. Theories such as social practice theory (27) and the normalization process theory (28) are some dominant approaches in the rural sociology for incorporating impacts of social contexts to understand behavior (29, 30). Also approaches exploring the role of cultural scripts (31), the concept of capital, habitus and field (32, 33) and the identity and lay-expert knowledge reflexivity are often applied for incorporating social contexts to understand behavior (29, 30).

Use of various psychosocial theories can help us to understand key constructs driving behaviors. Although review manuscripts regarding farmers' behavior have been published in the scientific literature (4, 34, 35), there is no comprehensive review assessing what, if any, psychosocial theories have been applied and what constructs or factors were identified.

### Comprehensive Frameworks and Tools for Behavior and Behavior Change

Given the complexity of behavior and behavior change, there have been attempts to develop comprehensive frameworks that can guide research, intervention design, and assist non-experts such as policymakers to understand human behavior. Two such widely cited frameworks are the social ecological framework or model (SEM) (36) and Behavior Change Wheel (BCW) (26).

The SEM is based on the previously developed ecological systems theory by Bronfenbrenner (36). SEM provides a holistic multilevel framework that considers the complex interplay among personal, interpersonal, and contextual environments to understand behavior (37). The SEM contextualizes behavior of individuals using various dimensions. These dimensions include personal (e.g., knowledge, attitudes, behavior), interpersonal (e.g., social networks, social supports), community (e.g., relationships among organizations/institutions), and public policies (e.g., local, state, and national laws) and how these factors interplay with each other (38). The SEM has been widely used for health promotion since the 1980s (39).

**Figure 1:**
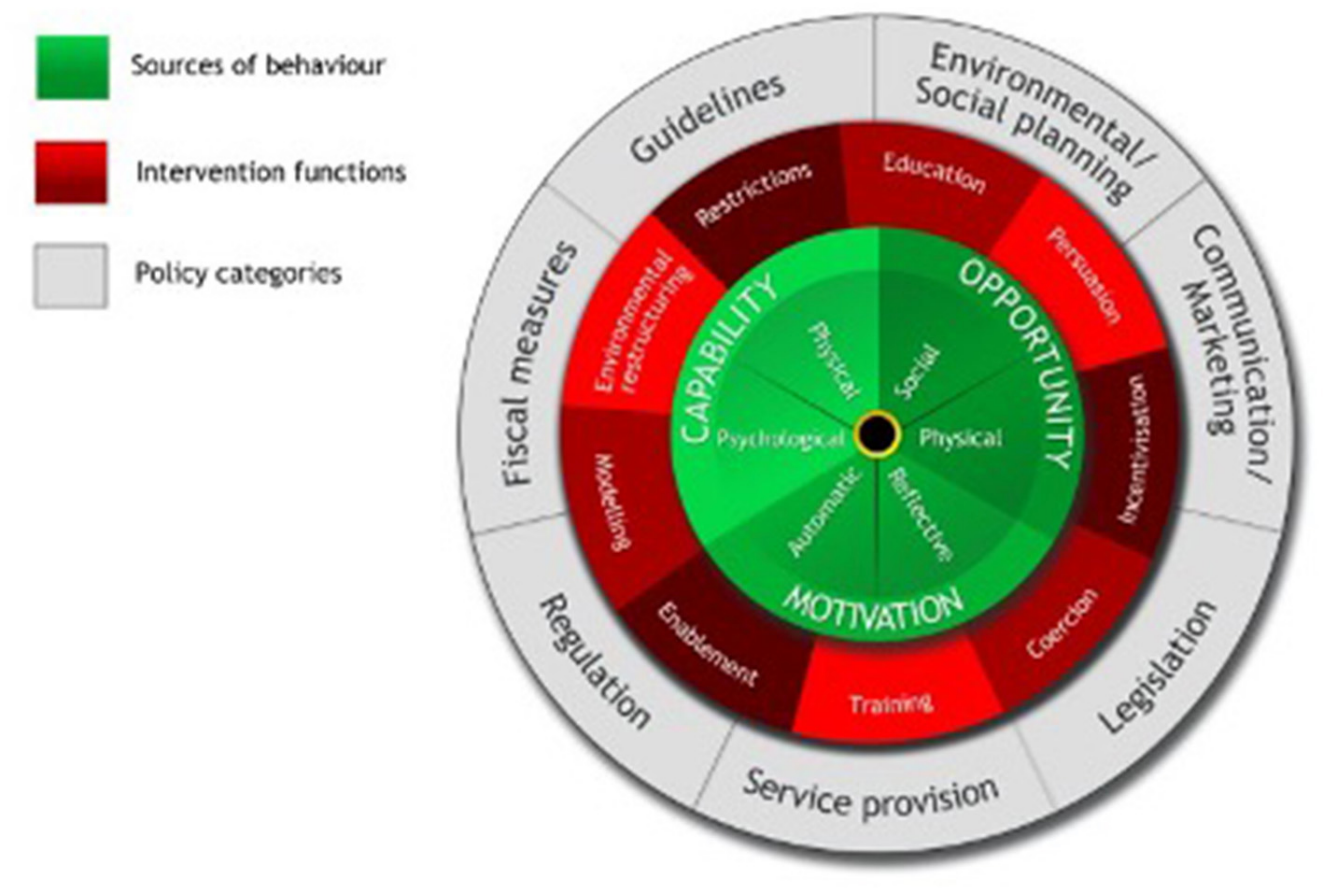
Behavior change wheel (26).

Whilst the purpose of the SEM is having broad inclusion of constructs that impact behavior, more recently developed frameworks tend to not only focus on identifying influences on behavior change, but also on understanding mechanisms of change and implementation of interventions. The BCW, which is linked to the Capability-Opportunity-Motivation (COM-B) model of behavior and is derived from human health research, is an example of such a framework (Figure 1). COM-B, designed by Michie et al. (26), consolidates the overlapping constructs of various theories. It provides a systematic way of characterizing interventions that enable their outcomes to be linked to mechanisms of action (12). The BCW is a synthesis of 19 health psychology theories and frameworks and includes the assumption that behavior is not only driven by beliefs and perception, but also by unconscious biases, mental shortcuts, and physical and contextual environments (12). The BCW consists of three layers: the hub of the wheel [the Capability-Opportunity-Motivation Behavior (COM-B) model], an intervention layer with nine functions, and a policy layer with seven categories (Figure 1). The hub of the wheel identifies the three constructs of behavior systems that could prove to be targets for intervention (12). “Capability” is subdivided into psychological capability and physical capability. “Opportunity” can be subdivided into social and physical opportunity, and “motivation” into automatic and reflective motivation. All three constructs (capability, opportunity, and motivation) are conceptualized as being essential for behavior (12). The BCW was successfully used in human health interventions to change human behavior [e.g., (40, 41)]. To successfully apply this method, it is crucial to be specific about the target group and its behavior.

The COM-B model and SEM enable exploring constructs that are not only related to the individual context but are also inclusive of broader contextual and social constructs. Therefore, by targeting interventions on various levels, we assume these frameworks to be more suitable for sustainable behavior change than frameworks that do not enable contextual and social constructs.

Understanding broader and complex behavioral structures is necessary before behavioral changes can be made, but also before key constructs of behavior can be incorporated into any animal disease prevention and control models. There is very limited work in the veterinary domain to design and test theory-informed behavior interventions (42).

In addition to choosing the appropriate theory or framework to successfully understand behavior, it is crucial to use robust design and data collection methodologies, including validity of questions or scales to measure constructs and/or behavior.

### Data Collection Approaches to Measure Attitudes, Beliefs, Perceptions, and Behaviors

#### Quantitative Methods

The purpose of quantitative research is to explain a phenomenon by the collection of numerical data that are analyzed by mathematical based methods and statistical approaches (43). Quantitative data help us to establish trends in the population and establish generalizability of the finding (44). Surveys are the most popular and widely used method to capture information on attitudes and behaviors. Most commonly, these surveys are deployed in a cross-sectional study design. Data on attitudes could be collected also using longitudinal study design where surveys and questionnaires can be used to track responses over time, e.g., by asking the same questions at various time points, or they can compare differences across space, e.g., by asking the same questions in different countries using, for example, the British Social Attitudes Survey (45) and European Social Survey (46). Finally, case-control studies could be also conducted to compare attitudes of two group. Attitudes are generally measured by a series of statements evaluated by Likert-scales, which uses five or six levels of agreement/disagreement. Another method used is the semantic differential scale, pairs of opposite concepts (e.g., strong/weak; democratic/authoritarian). The space between opposites is graded from a low e.g., 0 or 1 (expressing the lowest evaluation) to a high number e.g., 5 or 6 (representing the highest evaluation) (47). After developing and validating the scale, data analysis begins by a statistical or empirical validation with the collected quantitative data from a survey by employing statistical techniques to ensure construct reliability and validity. Examples are explanatory factor analysis (mainly done for new scales), confirmatory factor analysis (48), confirmatory composite analysis (utilizing structural equational modeling technique) (49), Cronbach Alpha (50) and item response theory (51). Further analysis linking these constructs and behavior is conducted using for example regression techniques and latent class models (10, 52).

#### Qualitative Methods

Qualitative approaches are most useful for providing rich contextual subjective information about people's feelings, emotions, perceptions, and attitudes. Sometimes, a qualitative approach is used as part of an empirical process of designing appropriate measurement scales. Common approaches to obtain qualitative data from study populations include in-depth interviews, focus groups, expert panels (53, 54) and ethnographies. Often, data collection occurs via audio recordings and these recordings are transcribed and analyzed. Thematic analysis, commonly employed for qualitative data analysis, could either be done deductively or inductively (55). In deductive analysis, themes or key categories are already defined before the analysis, e.g., script theory or Bourdieu concepts of field, capital, or COM-B model. An inductive approach is where categories are derived from the data, and new theories or hypotheses are developed. Grounded theory methodology (56), exclusively used for qualitative research, involves collecting and analyzing data (constant comparison) to formulate a theory.

Rich and thick descriptions are at the cornerstone of qualitative research; however, if there is no strong framework, the details may devolve into a story that is difficult to transfer and understand (16).

#### Mixed Methods

A mixed method combines elements of qualitative and quantitative research approaches for the purposes of breadth and depth of understanding, corroboration, and triangulation (57). It has been proposed that “triangulation,” which uses multiple data sources, helps increase the validity, strengths, and interpretative potential of a study (58). When using qualitative and quantitative methods to answer the same research question, emphasis should be on convergence, divergence, complementary and expansion of results (9, 59). Convergence means results from two methods being similar, whereas divergence means results from two methods being different (59). Complementary means results are different and not overlapping, and expansion refers to results having overlapping themes but non-overlapping interpretations (59, 60).

## Review: Sociological Research in Control of Cattle Diseases

### Search Strategy

PubMed and Web of Science were screened on 26 October 2020 for potentially relevant articles. We developed a search strategy consisting of relevant keywords describing the following themes: farmer, veterinarian, behavior/behaviour, perception, attitude, beliefs, and disease control. The broad themes were combined into a single query. A search containing the following words was conducted:

(farmer^*^or producer^*^or veterinarian^*^ *or* veterinary *or* vet *or* vets) *AND* (cattle *or* beef *or* dairy *or* cow^*^or calf *or* calves *or* heifer^*^) *AND* (belie^*^or behaviour^*^or behavior^*^or attitude^*^or perception^*^ *or* driver^*^or barrier^*^or enabler^*^or motivat^*^) *AND* (manag^*^or control^*^or implement^*^or uptake *or* prevent^*^ *or* use^*^or usage^*^).

As an additional analysis, influences of veterinarians on farmers were explored, as veterinarians are deemed to be very important in farmers' decision making. Therefore, studies focusing on veterinarians' behavior were also assessed for eligibility. Although no limits on publication date nor language were applied during our initial screening, only English and Dutch articles were considered for full-text reviewing. Queries were adapted to database-specific terms, as deemed necessary. Using these search criteria, 6,667 manuscripts were identified in both databases, and after duplicate removal, abstracts of 5,134 manuscripts were screened. For inclusion in the final review, we formulated additional criteria: (1) the subject of the study had to be related to prevention or control of cattle disease; and (2) the study should have a focus on behavioral key constructs of farmers. Finally, reference lists from all articles included in this review were reviewed for potential inclusion (Table 1).

**Table 1:** Inclusion criteria.

**CRITERIA**
**Farmers and veterinarians**
**Language: English and Dutch**
**Subject: related to prevention and control of cattle disease**
**Focus: behavioral key constructs**


**Figure 2:**
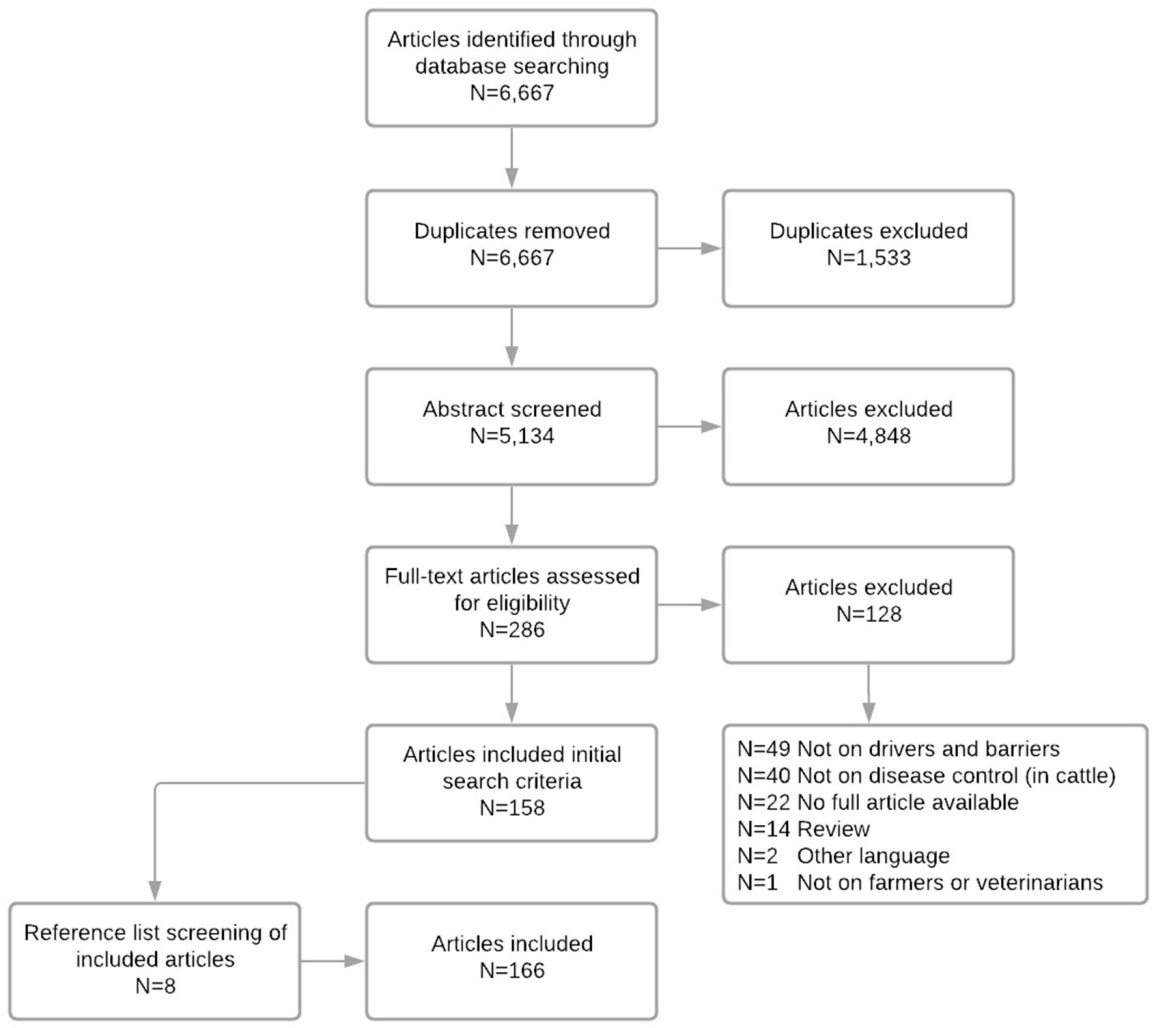
Prisma flow-chart of included articles.

Eight additional manuscripts, identified by screening reference lists of included manuscripts, were eligible according to the inclusion criteria and were included in the full text screening. After applying all criteria, 166 manuscripts remained for full text screening (Figure 2). The web-based software Covidence^®^ (61) was used for screening and data extraction. Screening and data extraction was conducted by the first author and discussed in detail with author JK after each phase. The 166 identified manuscripts are included in the Supplementary Material.

## Findings on Farmers' Behavior in Relation to Disease Control on Cattle Farms

### Themes of Disease Control and Countries

The search criteria led to the inclusion of 166 manuscripts, of which 133 studied farmers' behavior, 13 a combination of farmers' and veterinarians' behavior, and 20 studied only veterinarians' behavior. Results of the 20 studies on veterinarians' behavior will be presented independent from all other results. In the 146 remaining studies, 24 themes of disease prevention and control were identified, with the most intensively studied theme being the application of biosecurity measures (*n* = *22; 15%*) and bovine paratuberculosis (Johne's disease) (*n* = *22; 15%*), followed by behavior related to use of antimicrobials and prescriptions for antimicrobials by farmers and veterinarians (*n* = *20; 14%*), and mastitis (*n* = *19; 13%*) (Table 2). The relatively large number of studies on antimicrobial use and antimicrobial resistance was attributed to emergence of antimicrobial resistance in human and animal health (62), and regulations to decrease antimicrobial use (63).

**Table 2:** Manuscripts (*N* = 146) describing farmers' behavior regarding animal disease prevention and control.

**Theoretical underpinning^a^**	***N***	**Analytical methodology^a^**	***N***	**Themes**	***N***	**Data gathering**	***N***
None	87	Thematic analysis	33	Biosecurity	22	Quantitatively	74
Theory of planned behavior	21	Non-parametric tests	25	Johne's disease/Paratuberculosis	22	Qualitatively	43
Grounded theory	11	Logistic regression analysis	20	Antimicrobial use	20	Mixed method	29
Theory of reasoned action	7	Descriptive^b^	18	Mastitis	19		
Health belief model	5	Principle component analysis	11	Disease control^c^	12		
Behavioral economics theory	3	Content analysis	8	Foot lesions	8		
Mental model	1	Structural equation modeling	8	Foot and mouth disease	6		
Social ecology framework	2	Cronbach's alpha	5	Health management	5		
Transtheoretical model	2	Factor analysis	6	Vaccinations	5		
Agency theory	1	Student's *t*-tests	5	Bluetongue	4		
Appreciative inquiry	1	Linear regression analysis	3	Bovine viral diarrhea	4		
Bourdieu	1	Negative binomial regression model	3	Tick borne diseases	4		
Design thinking process	1	Q-methodology	3	Helminths	3		
Diff-con theory	1	Adaptive conjoint analysis	2	Calf mortality	2		
Fogg behavior model	1	Bayesian network analysis or approach	2	Anthrax	1		
Pike's model	1	4-step methodology	2	Brucellosis	1		
Prospect theory	1	Biographical narrative interpretive method	2	Bovine dermatophilosis	1		
Precaution adoption process model	1	Open coding	3	Contagious bovine pleuropneumonia	1		
Social identity theory	1	Probit analysis	3	*Escherichia coli*	1		
Theory of change	1	Cluster analysis	2	East coast fever	1		
Theory of knowledge	1	Generalized linear mixed model	2	Hydatid disease	1		
Trigger change model	1	Axial coding	1	Mange control	1		
		Analytic induction analysis	1				
		Latent class analysis	1				
		Hierarchical clustering	1				
		Roter interaction analysis system	1				
		Time series analysis	1				
		Naturalistic paradigm	1				
		Paradigmatic model	1				
		Scenario-based mapping methodology	1				
		Social network analysis	1				
		Monte Carlo simulation	1				
		Interval regression analysis	1				
		Econometric adoption model	1				
		Ordered multinomial regression model	1				

a *One manuscript can be based on several theories or methodologies*.

b *Only when no other methodology but descriptive statistics was mentioned*.

c *Disease control consists of topics such as herd health management, surveillance programs and adoption of veterinarian's advice*.

**Figure 3:**
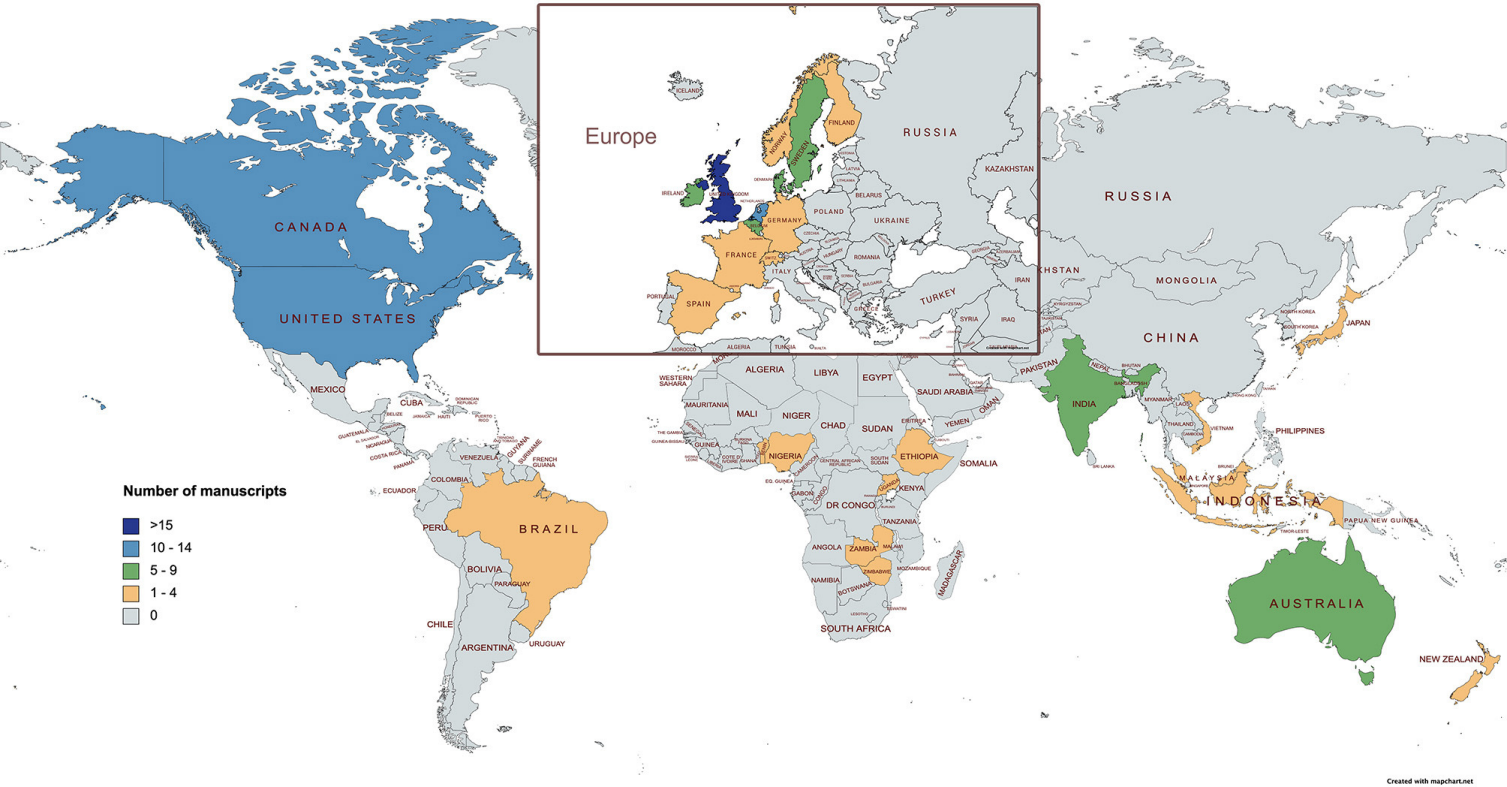
Worldwide distribution of manuscripts on farmers' behavior concerning cattle disease prevention and control.

Manuscripts that studied farmers' behavior within cattle disease prevention and control were identified from 27 countries worldwide (Figure 3). Researchers from the United Kingdom published the most manuscripts (*n* = *35*), followed by researchers from the Netherlands (*n* = *15*), Canada (*n* = *13*), and the USA (*n* = *13*). Researchers from six African countries published a total of 11 manuscripts; researchers from five Asian countries published nine manuscripts, and South American researchers published one manuscript originating from Brazil. In Europe, research in this area was mainly done by researchers from north-western European countries (Figure 3). Specifically, European countries were Belgium (*n* = 6), Denmark (*n* = 5), France (*n* = 4), Germany (*n* = 3), Ireland (*n* = 6), Spain (*n* = 3), Sweden (*n* = 9), Switzerland (*n* = 4) and a combination of the Nordic countries (*n* = 1). Some publications were a result of a collaboration of researchers and data from more than one country.

### Trend Over Time, Theoretical Underpinning and Methodological Approaches

**Figure 4:**
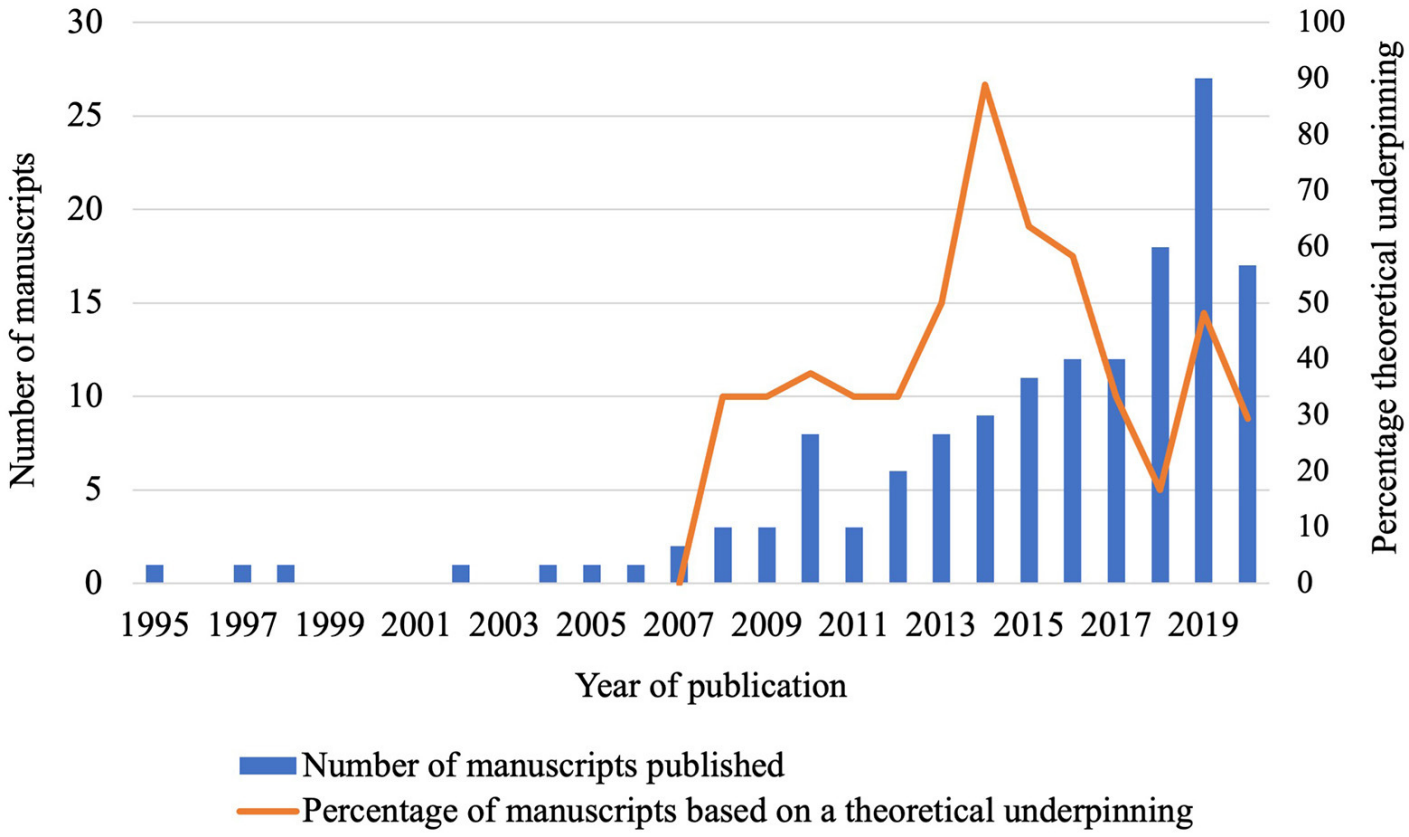
Manuscripts about farmers' behavior regarding cattle diseases published (Pubmed or Web of Science) between 1995–2020, and the subset based on a theoretical underpinning.

The first manuscript identified was published in 1995 by Australian authors (64). The increased appreciation of social science studies in the veterinary field is substantiated by the total number of manuscripts published between 1995–2009 (*n* = 15), 2010–2014 (*n* = 34) and 2015–2019 (*n* = 97) (Figure 4; IRR = 2.6, *P* < 0.001).

Although application of social sciences in farmers' behavior studies has increased, between 2008 and 2020, the average percentage of manuscripts that applied a theory varied from 17 to 89% per year (Figure 4). Overall, 58% of the published studies lacked any theoretical underpinning. Therefore, there is a lack of theory-informed tools to research in farmers' behavior related to cattle disease prevention and control. The percentage of studies based on theory increased from 2012 until 2014, relative to the period until 2011 (OR = 3.5, *P* = 0.04). However, in the years thereafter (2015–2018), this percentage tended to decrease compared to 2012–2014 (OR = 0.42, *P* = 0.09). The number of manuscripts based on theory in 2019–2020 did not differ from 2015 to 2018 (*P* = 0.90) (Figure 4). Not all manuscripts of 2020 could be included in our review (search date October 26th), but it seems unlikely that the current percentage of 29% will increase substantially when the search term is used again. Reasons for the declining percentage of theoretical underpinned veterinary research remains unclear. It might be related to challenges of interdisciplinary research, and that most research on theories is published in sociological journals, which likely have limited veterinary readership. Presumably dissemination of developments in social science research to veterinary research is limited or at least suboptimal. Further, use of inductive approaches are a common feature of qualitative studies and half (51%) of the qualitative studies in the review were identified as exploratory. However, in studies using quantitative or mixed methods, 61 and 62%, respectively, lacked use of a theory.

The most frequent theoretical underpinning was based on the TPB (14%). The TPB “surge” was also noticed in the human health studies but occurred a decade before the TPB “surge” in farmers' behavior studies, with the main increase between 1997 and 2012 (21). A critical limitation of the TPB is that it is unable to explain sufficient variability in the data (65). For example, in human health studies, the TPB explained 39% of the variance in intentions but only accounted for 27% of human behavior variance (66). Another concern about the TPB is its utility, as it fails in the primary function of a theory; namely, it does not accurately communicate accumulated empirical evidence and therefore does not enable practitioners to develop helpful interventions (Sniethotta et al., 2014). The TPB is more useful for predicting self-assessed behavior than objective measurement of behavior and predicts behavior better in the short vs. long term (21). In human health studies, there is a shift to frameworks that are composite of theory domain frameworks and are better for understanding and changing behavior, such as the COM-B model (26).

The second most used method in these studies was the grounded theory (8%). The overall aim in grounded theory is to generate theories inductively by collecting data about a phenomenon, identifying key elements, and categorizing the relationship of those elements to each other (67). However, only 4 of the 11 manuscripts based on grounded theory ultimately mentioned the development of own theory, indicating failure to ensure that the analysis moved beyond narrative description to generate theoretical concepts (67). This finding illustrates that the theoretical and philosophical underpinning of grounded theory was often not completely understood.

Health psychology theories and constructs are validated in human health research, but there is limited evidence of measurement scale validation and very few have been statistically validated in the context of animal health (68–70). In the reviewed work, there was a lack of confirmatory factor analysis testing theories and constructs. Human health and animal health environments differ considerably. Although farmers make decisions concerning themselves, similar to human health, they also make decisions that affect their animals and business. Therefore, it might be that these theoretical constructs may have even less explanatory value in the context of management of animal health. This hypothesis needs to be explored further.

### Methods of Data Collection

Data were collected quantitatively in 51% of the studies, while in 29% of the studies, data were collected qualitatively. A mixed approach was used for the remaining 20% (Table 2). Questions to determine farmers' perceptions, beliefs, and attitudes were often measured with 5 or 6-point Likert scales. For countries that published ≥5 manuscripts, quantitative or mixed approaches were more often used, except for India and Canada, where data were mostly collected qualitatively (Table 3). Researchers from Scandinavian countries and the United Kingdom used qualitative and quantitative methods with approximately equal frequency.

**Table 3:** Manuscripts (%) with quantitative, qualitative or a mixed method approach to collect data per country^a^ that published at least five manuscripts.

**Data gathering**	**USA**	**AUS**	**BE**	**CA**	**DK**	**IND**	**IRE**	**NL**	**SE**	**UK**
Quantitatively	54	63	67	31	20	40	50	71	44	43
Qualitatively	15	25	17	38	40	60	33	14	33	34
Mixed method	31	13	17	31	40	0	17	14	22	23

a *USA, United States of America; AUS, Australia; BE, Belgium; CA, Canada; DK, Denmark; IND, India; IRE, Ireland; NL, the Netherlands; SE, Sweden; UK, United Kingdom*.

### Key Behavioral Constructs

To illustrate the complexity of factors impacting farmers' behavior, and the consequences of the lack of theoretical underpinning, key behavioral constructs of farmers' behavior related to cattle disease prevention and control were determined per study and summarized according to the SEM and the BCW of the COM-B model.

#### Social-Ecological Model

Each of the constructs identified in our review was assigned to the personal, interpersonal, and contextual environments of SEM (Table 4). Mainly, constructs were related to individual influences (*N total mentioned* = *608)*. Determinants related to these constructs are individual beliefs on cost-benefits, perceived risk (perceived), knowledge, and perceived control, but also beliefs about what others think. Constructs related to the interpersonal and contextual influences were mentioned less often (173 and 172 times, respectively). Constructs of the interpersonal influence consisted mainly of relationships with the veterinarian, peers, family, and employees. Constructs of the contextual influences were mentioned less frequently, but there was a huge variety of contextual influences in relation to farmers' decisions. This finding was not surprising, as contextual factors are likely to vary among countries with distinct legislations, industry initiatives, rewards and penalties related to disease control.

**Table 4:** Summary of farmers' behavior constructs described in 146 peer-reviewed studies related to cattle disease prevention and control and indexed in Pubmed and Web of Science.

**Personal**		**Interpersonal**		**Contextual**	
**Determinant**	***N***	**Determinant**	***N***	**Determinant**	***N***
Cost-benefits belief	107	Farmer-veterinarian relationship	83	Farmer-government or industry influence	39
Perceived risk	102	Farmer-farmer relationship	38	Legislation	25
(Perceived) knowledge	100	Normative beliefs	34	Incentives	27
Perceived control	75	Farmer-family relationship	7	Farm limitations	23
(Perceived) efficacy measures	53	Farmer-employee relationship	7	Culture	12
Previous experience	41	Magazines	1	Production type	10
(Perceived) time	33	Farmer-land owner relationship	1	Guidelines	4
Job satisfaction	21	Retailer-consumer relationship	1	Logistics	4
Perceived practicality	21	Science-public relationship	1	Marketing by pharmaceuticals	3
Age of the farmer	16			Understandable label on drugs	3
Educational level of farmer	11			Access to diagnostic laboratories	2
Habits	7			Membership in health scheme	2
Emotion	8			Milk price	2
Gender of farmer	6			Profusion of informal prescribers	2
Jealousy	2			Poverty	2
Personality of the farmer	2			Accreditation issues	1
Ability to physically change	1			Access to technology	1
Perceived consumer education	1			Availability of the drug	1
Coping capacity	1			Bank loans	1
				Contractual restrictions	1
				Country	1
				Inadequate transportation	1
				Institutional failure	1
				Resources	1
				Pharmaceutical sales representative	1
				Water availability	1
				Walking long distances	1
**Total**	608		173		172

Although most reported constructs belonged to the personal environment, it cannot be concluded that these factors are most important. Most likely, constructs belonging to the interpersonal and contextual environment were not extensively studied due to the limitations mentioned earlier in research methods used to study farmers' behavior. A drawback from focusing mainly on individual influences on behavior, is that understanding of more complicated and complex social, economic, and political influences remains unexplored (8, 71).

#### COM-B

Each of the constructs identified in our review was assigned to one of the COM-B components: physical capability, psychological capability, physical opportunity, social opportunity, reflective motivation, or automatic motivation (Table 5). The large number of constructs identified illustrated the complexity of factors impacting farmers' behavior.

**Table 5:** Summary of constructs of farmers' behavior described in 146 reviewed studies related to cattle disease control mapped in a COM-B model and indexed in Pubmed or Web of Science.

**Capability**		**Opportunity**		**Motivation**	
**Psychological**	**Physical**	**Social**	**Physical**	**Reflective**	**Automatic**
Knowledge	Age	Farmer-veterinarian relationship	Cost-benefits	Perceived risk	Emotion
Personality	Gender	Farmer-farmer relationship	Resources	Perceived control	Habits
Coping capacity		Family-family relationship	Legislation	Previous experience	Jealousy
Ability to change		Farmer-employee relationship	Incentives	(Perceived) efficacy of measures	
Education		Farmer-land owner relationship	Guidelines	Perceived practicality	
		Retailer-consumer relationship	Milk price	Job satisfaction	
		Science-public relationship	Accreditation issues	Belief of marketing by pharmaceuticals	
		Farmer-government or industry influence	Availability of the drug	(Perceived) time	
		Culture	Logistics	Normative beliefs	
		Media	Access to diagnostic laboratories	Perceived consumer education ‘Good farmer identity’	
		Pharmaceutical sales representative	Production type		
			Farm limitations		
			Membership in health scheme		
			Bank loans		
			Country		
			Poverty		
			Water availability		
			Long distances to walk		
			Access to technology		
			Contractual restrictions		
			Transportation		

The majority of the manuscripts indicated multiple components of the relationship between farmers and veterinarians (social opportunity). Moreover, the perceived risk of farmers, their (perceived) knowledge, perceived control (perceived), cost-benefits, relationship with peers and advisors, and previous experience were recurrent determinants that were reported to influence farmers' decision-making process. Most determinants were related to reflective motivation or physical opportunities (Table 5). Opportunities can influence motivation, emphasizing the importance of assessing interconnectedness of these constructs.

As illustrated from the BCW, interventions only related to farmers' ‘education’ to drive behavior change will have very limited impact. Given the range of constructs impacting behavior change, there is more need to test a wide range of interventions including persuasion, and incentivization, changes to environment/social contexts, marketing and communication (12). Interestingly, although constructs of automatic motivation have a substantial influence on human behavior, very few determinants have been identified in veterinary studies (10).

Also, although the results of mapping farmers' behavior based on the COM-B model indicated a wider range of constructs, we cannot state which component had the most influence, due to variations in study designs and because most studies were cross-sectional.

### Influence of the Veterinarian

In 73 of the farmers' behavioral studies, originating from 18 countries, components of the farmer-veterinarian relationship were explored. The likely large influence of veterinarians on farmers' behavior was also supported by the reviewed literature; veterinarians are often regarded as the primary source of information on animal health and disease control (68, 72–76). It is, therefore, important to consider influences of veterinarians on farmers' decision-making processes. To achieve a better understanding of veterinarians' behavior, a subset of 20 veterinarians' behavior studies was explored. Veterinarians' behavior was studied in 12 countries, including the United Kingdom (*N* = 4), the USA, Canada, the Netherlands, Sweden, Switzerland (*N* = 2 per country), Belgium, Ireland, Australia, Argentina, Italy, and Peru (*N* = 1 per country). The first manuscript was published in 2009 by US authors (77). Themes studied were mainly related to antimicrobial use or attitude toward antimicrobial resistance (39%) and perception of on-farm biosecurity (28%).

More often, constructs mentioned were related to the personal environment (*N total mentioned* = *103*), than to the interpersonal or contextual environment (*N total mentioned* = *27 and 27, respectively)*. There was a limited list of contextual factors influencing veterinarians' behavior, perhaps due to harmonization of practice standards or accreditation requirements under which veterinarians operated (Table 6). Personal determinants listed include veterinarians' own beliefs and perceptions as well as how they perceived farmers. Based on the evidence, we inferred that how veterinarians frame barriers to disease control differed from how farmers frame these barriers; unlike farmers, veterinarians put more emphasis of individual and interpersonal factors, as was apparent in the review (8). To adapt to changing policy and practice environments, veterinary practitioners must more effectively blend scientific and evidence-based approaches to veterinary care with the pragmatic concerns facing farmers by fully understanding farmers' determinants of disease control (8, 78).

**Table 6:** Summary of veterinary behavior constructs described in 20 peer-reviewed studies related to cattle disease prevention and control and indexed in Pubmed or Web of Science.

**Personal**		**Interpersonal**		**Contextual**	
**Determinant**	***N***	**Determinant**	***N***	**Determinant**	***N***
Perceived risk of farmer by veterinarian	11	Farmer-veterinarian relationship	16	Legislation	6
Perceived risk of veterinarian	13	Vet-vet relationship	4	Farm limitations	4
(Perceived) knowledge of farmer by veterinarian	8	Normative beliefs	2	Guidelines	3
(Perceived) knowledge of veterinarian	11	Vet-non-vet prescribers' relationship	2	Vet-government or industry influence	3
Cost-benefits of farmer by veterinarian	10	Vet-pharmaceutical relationship	1	Competition	2
Cost-benefits of veterinarian	4	Farmer-farmer relationship	1	Size and type of veterinary practice	2
Previous experience of farmer by veterinarian	1	Magazines	1	Access to diagnostic laboratories	1
Previous experience of veterinarian	10			Availability of the drug	1
(Perceived) time of farmer by veterinarian	4			Client confidentiality	1
(Perceived) time of veterinarian	6			Country	1
Perceived control of farmer by veterinarian	2			Hierarchical structure of veterinarian practices	1
Perceived control of veterinarian	6			Incentives	1
(Perceived) efficacy measures by veterinarian	5			Insurance	1
Educational level of farmer by veterinarian	1				
Educational level of veterinarian	2				
Habits of farmer	2				
Habits of vet	1				
Age of the farmer	1				
Age of the veterinarian	2				
Perceived practicality for veterinarian	1				
Personality of the veterinarian	1				
Privacy of the farmer	1				
**Total**	103		27		27

## Conclusion and Recommendations

The aims of this review were to explore the use of psychosocial theory, sociological approaches, and analytical methodologies in research studying farmers' behavior. We focused on the context of cattle disease control, determined key constructs and where they mapped in recently developed behavior change frameworks. Our review indicated key gaps in the current published research. Firstly, there was limited use of theoretical underpinning or explicit theory when aiming to understand farmers' behavior. Furthermore, there was little evidence to validate any of the determinants related to farmers' behavior in cattle disease prevention and control or any interventions that lead to behavior change. To successfully change farmers' behavior, it is essential to understand influences on behavior, behavioral change mechanisms and implementation of interventions. Moreover, these understandings are crucial for knowing which theories are effective in veterinary epidemiology and ultimately for incorporating a human behavioral factor in infectious disease modeling. The explicit use of theory can promote understanding of complex behavioral structures. Additionally, our review indicated the complexity of constructs with effects at individual, interpersonal and contextual levels related to capability, motivation, and opportunity. Published studies have mostly focused on constructs at individual levels. Therefore, it is recommended that for a sustainable behavior change, studies should include wider constructs at individual, interpersonal and contextual levels. Also, interdisciplinary research involving persons specialized in applying social science techniques and associated analytical methods are key. The resulting constructs could subsequently be linked to interventions and be the first step toward theory-driven evidence-based interventions to influence farmers' behavior for disease control.

## Author Contributions

IS-B provided input on the initial idea and her expertise throughout the project and helped in writing the manuscript. JB and MG provided input on the inital idea and study design. HB provided input on writing the manuscript. CR provided her expertise on behavioral research and helped in writing the manuscript. JK had a supervisory role over MB while she visited the University of Nottingham and thereafter. JK provided the initial idea, her expertise throughout the project, and contributed to writing the manuscript. All authors contributed to in the writing of this manuscript, have read the final version, and agreed upon its content.

## Funding

This article is based upon work from COST Action SOUND control [CA17110], supported by COST (European Cooperation in Science and Technology). This study was supported by the Industrial Research Chair in Infectious Diseases of Dairy Cattle, funded by Canada's Natural Sciences and Engineering Research Council (NSERC) Industrial Research Chair Program (Ottawa, ON, Canada), with industry contributions from Alberta Milk (Edmonton, AB, Canada), the Dairy Farmers of Canada (Ottawa, ON, Canada), Westgen Endowment Fund (Milner, BC, Canada), the BC Dairy Association (Burnaby, BC, Canada), Canadian Dairy Network (Guelph, ON, Canada), CanWest DHI (Guelph, ON, Canada), SaskMilk (Regina, SK, Canada), Dairy Farmers of Manitoba (Winnipeg, MB, Canada) and MSD Animal Health (Kirkland, QC, Canada). Figure 1 was originally published in Michie et al. (26), part of Springer Nature Group. Copyright BWC Wheel and used with permission.

## Conflict of Interest

The authors declare that the research was conducted in the absence of any commercial or financial relationships that could be construed as a potential conflict of interest.

## Publisher's Note

All claims expressed in this article are solely those of the authors and do not necessarily represent those of their affiliated organizations, or those of the publisher, the editors and the reviewers. Any product that may be evaluated in this article, or claim that may be made by its manufacturer, is not guaranteed or endorsed by the publisher.
