# Supplementary material for: Understanding Farmers' Behavior and Their Decision-Making Process in the Context of Cattle Diseases: A Review of Theories and Approaches
Source: Front Vet Sci. 2021 Dec 2;8:687699. doi: 10.3389/fvets.2021.687699 (PMC8674677; doi:10.3389/fvets.2021.687699)
Supplement: Supplementary file 1 [file Table_1.docx]

**Supplementary Materials**

**Manuscripts included in the review**

Adehan SB, Adakal H, Gbinwoua D, Yokossi D, Zoungrana S, Toé P, et al. West African Cattle Farmers’ Perception of Tick-Borne Diseases. Ecohealth. 2018;15(2):437–49. doi: 10.1007/s10393-018-1323-8.

Alhaji NB, Aliyu MB, Ghali-Mohammed I, Odetokun IA. Survey on antimicrobial usage in local dairy cows in North-central Nigeria: Drivers for misuse and public health threats. PLoS One 2019;14(12):e0224949. https://doi.org/10.1007/s11250-017-1445-y.

Alhaji NB, Isola TO. Pastoralists’ knowledge and practices towards clinical bovine dermatophilosis in cattle herds of North-Central Nigeria: the associated factors, burden and economic impact. Trop Anim Health Prod. 2018;50(2):381–91. doi: https://doi.org/10.1007/s11250-017-1445-y.

Amenu K, Szonyi B, Grace D, Wieland B. Important knowledge gaps among pastoralists on causes and treatment of udder health problems in livestock in southern Ethiopia: Results of qualitative investigation. BMC Vet Res. 2017;13(1):303. doi: 10.1186/s12917-017-1222-1.

Azbel-Jackson L, Heffernan C, Gunn G, Brownlie J. Exploring the role of voluntary disease schemes on UK farmer bio-security behaviours: Findings from the Norfolk-Suffolk Bovine Viral Diarrhoea control scheme. PLoS One. 2018;13(2):e0179877. doi: 10.1371/journal.pone.0179877.

Belage E, Croyle SL, Jones-Bitton A, Dufour S, Kelton DF. A qualitative study of Ontario dairy farmer attitudes and perceptions toward implementing recommended milking practices. J Dairy Sci. 2019;102(10):9548–57. doi: 10.3168/jds.2018-15677

Benjamin LA, Fosgate GT, Ward MP, Roussel AJ, Feagin RA, Schwartz AL. Attitudes towards biosecurity practices relevant to Johne’s disease control on beef cattle farms. Prev Vet Med. 2010;94(3–4):222–30. doi: 10.1016/j.prevetmed.2010.01.001.

Bennett R, & Balcombe K. Farmers’ willingness to pay for a tuberculosis cattle vaccine. J. Agric. Econ. 2012;63(2): 408-424. doi: 10.1016/j.tvjl.2013.09.068.

Bennett RM, Barker ZE, Main DCJ, Whay HR, Leach KA. Investigating the value dairy
 farmers place on a reduction of lameness in their herds using a willingness to pay
 approach. Vet J. 2014;199(1):72–5. doi:
 <https://doi.org/10.1111/j.1477-9552.2011.00330.x>

Bhattacharyya A, Harris TR, Kvasnicka WG, Veserat GM. Factors influencing rates of adoption of trichomoniasis vaccine by Nevada range cattle producers. J Agric Resour Econ. 1997;22(1):174–90.

Bock R, Blight G, Kingston T, de Vos A. A survey of cattle producers in the *Boophilus microplus* endemic area of Queensland to determine attitudes to the control of and vaccination against tick fever. Aust Vet J. 1995;72(3):88–92. doi: <https://doi.org/10.1111/j.1751-0813.1995.tb15015.x>

Brennan ML, Christley RM. Cattle producers’ perceptions of biosecurity. BMC Vet Res. 2013;9:71. doi: 10.1186/1746-6148-9-71

Brennan ML, Wright N, Wapenaar W, Jarratt S, Hobson-West P, Richens IF, et al. Exploring attitudes and beliefs towards implementing cattle disease prevention and control measures: A qualitative study with dairy farmers in Great Britain. Animals. 2016;6(10). doi: 10.3390/ani6100061

Bronner A, Hénaux V, Fortané N, Hendrikx P, Calavas D. Why do farmers and veterinarians not report all bovine abortions, as requested by the clinical brucellosis surveillance system in France? BMC Vet Res. 2014;10:93. doi: 10.1186/1746-6148-10-93

Brook RK, McLachlan SM. Factors influencing farmers’ concerns regarding bovine tuberculosis in wildlife and livestock around Riding Mountain National Park. J Environ Manage. 2006;80(2):156–66. doi: 10.1016/j.jenvman.2005.08.022

Broughan JM, Maye D, Carmody P, Brunton LA, Ashton A, Wint W, et al. Farm characteristics and farmer perceptions associated with bovine tuberculosis incidents in areas of emerging endemic spread. Prev Vet Med. 2016;129:88–98. doi: 10.1016/j.prevetmed.2016.05.007

Brownlie TS, Holmes I, Delahunty H, Salmon S, Hunnam JC. Perceptions of anthrax in livestock from Victorian dairy farmers in the Goulburn-Murray region of Victoria, Australia. Aust Vet J 2019;97(9):333–5. doi: 10.1111/avj.12844

Bruijnis M, Hogeveen H, Garforth C, Stassen E. Dairy farmers’ attitudes and intentions
 towards improving dairy cow foot health. Livest Sci. 2013;155(1):103–13.

Cattaneo AA, Wilson R, Doohan D, LeJeune JT. Bovine veterinarians’ knowledge, beliefs, and practices regarding antibiotic resistance on Ohio dairy farms. J Dairy Sci. 2009;92(7):3494–502. doi: 10.3168/jds.2008-1575

Charlton K, Robinson PA. A qualitative investigation of the attitudes and practices of farmers and veterinarians in wales regarding anthelmintic resistance in cattle. Vet Ital. 2019;55(4):327–37. doi: 10.12834/VetIt.1848.9845.3

Chauhan AS, George MS, Chatterjee P, Lindahl J, Grace D, Kakkar M. The social biography of antibiotic use in smallholder dairy farms in India. Antimicrob Resist Infect Control. 2018;7(1):60. doi: 10.1186/s13756-018-0354-9

Chauhan AS, George MS, Lindahl J, Grace D, Kakkar M. Community, system and policy level drivers of bovine tuberculosis in smallholder periurban dairy farms in India: A qualitative enquiry. BMC Public Health. 2019;19(1):301. doi: 10.1186/s12889-019-6634-3

Christley RM, Robinson SE, Moore B, Setzkorn C, Donald I. Responses of farmers to introduction in England and Wales of pre-movement testing for bovine tuberculosis. Prev Vet Med. 2011;100(2):126–33. doi: 10.1016/j.prevetmed.2011.02.005

Ciaravino G, Espluga J, Casal J, Pacios A, Mercader I, Allepuz A. Profiles of opinions among farmers and veterinarians towards the Tuberculosis Eradication Programme in cattle in Spain. Prev Vet Med 2020;176:104941. doi: 10.1016/j.prevetmed.2020.104941

Cousin ME, Härdi-Landerer MC, Völk V, Bodmer M. Control of Staphylococcus aureus in dairy herds in a region with raw milk cheese production: Farmers’ attitudes, knowledge, behaviour and belief in self-efficacy. BMC Vet Res. 2018;14(1):46. doi: 10.1186/s12917-018-1352-0

Cross P, Williams P, Edwards-Jones G. Differences in the perceptions of farmers and veterinary surgeons about the efficacy of mitigation strategies for controlling bluetongue. Vet Rec 2009;165(14):397–403. doi: 10.1136/vr.165.14.397

Damiaans B, Sarrazin S, Heremans E, Dewulf J. Perception, motivators and obstacles of biosecurity in cattle production. Vlaams Diergeneeskd Tijdschr. 2018;87(3): 150-163

Delgado AH, Norby B, Dean WR, McIntosh WA, Scott HM. Utilizing qualitative methods in survey design: Examining Texas cattle producers’ intent to participate in foot-and-mouth disease detection and control. Prev Vet Med. 2012;103(2–3):120–35. doi: 10.1016/j.prevetmed.2011.09.012

Delgado AH, Norby B, Scott HM, Dean W, McIntosh WA, Bush E. Distribution of cow-calf producers’ beliefs about reporting cattle with clinical signs of foot-and-mouth disease to a veterinarian before or during a hypothetical outbreak. Prev Vet Med. 2014;117(3–4):505–17. doi: 10.1016/j.prevetmed.2014.09.011

Delgado AH, Norby B, Scott HM, Dean W, McIntosh WA, Bush E. Distribution of cow-calf producers’ beliefs regarding gathering and holding their cattle and observing animal movement restrictions during an outbreak of foot-and-mouth disease. Prev Vet Med. 2014;117(3–4):518–32. doi: 10.1016/j.prevetmed.2014.09.010

Denis-Robichaud J, Kelton DF, Bauman CA, Barkema HW, Keefe GP, Dubuc J. Canadian dairy farmers’ perception of the efficacy of biosecurity practices. J Dairy Sci. 2019;102(11):10657–69. doi: 10.3168/jds.2019-16312

Derks M, van de Ven LMA, van Werven T, Kremer WDJ, Hogeveen H. The perception of veterinary herd health management by Dutch dairy farmers and its current status in the Netherlands: A survey. Prev Vet Med. 2012;104(3–4):207–15. doi: 10.1016/j.prevetmed.2011.12.019

Doidge C, Hudson C, Lovatt F, Kaler J. To prescribe or not to prescribe? A factorial survey to explore veterinarians’ decision making when prescribing antimicrobials to sheep and beef farmers in the UK. PLoS One. 2019;14(4):e0213855. doi: 10.1371/journal.pone.0213855

Dutton-Regester KJ, Wright JD, Rabiee AR, Barnes TS. Understanding dairy farmer intentions to make improvements to their management practices of foot lesions causing lameness in dairy cows. Prev Vet Med. 2019;171:104767. doi: 10.1016/j.prevetmed.2019.104767

Easton S, Pinchbeck GL, Bartley DJ, Hodgkinson JE & Matthews JB. A survey of experiences of UK cattle and sheep farmers with anthelmintic prescribers; are best practice principles being deployed at farm level?. Prev Vet Med. 2018;155:27-37. doi: https://doi.org/10.1016/j.prevetmed.2018.04.009

Ekakoro JE, Caldwell M, Strand EB, Okafor CC. Drivers of antimicrobial use practices among Tennessee dairy cattle producers. Vet Med Int. 2018;2018:1836836. doi: 10.1155/2018/1836836

Ekakoro JE, Caldwell M, Strand EB, Okafor CC. Drivers, alternatives, knowledge, and perceptions towards antimicrobial use among Tennessee beef cattle producers: A qualitative study. BMC Vet Res. 2019;15(1):16. doi: 10.1186/s12917-018-1731-6

Ekakoro JE, Caldwell M, Strand EB, Strickland L, Okafor CC. A survey of antimicrobial use practices of Tennessee beef producers. BMC Vet Res. 2019;15(1):222. doi: 10.1186/s12917-019-1978-6

Elbers ARW, De Koeijer AA, Scolamacchia F & Van Rijn PA. Questionnaire survey about the motives of commercial livestock farmers and hobby holders to vaccinate their animals against Bluetongue virus serotype 8 in 2008–2009 in the Netherlands. Vaccine. 2010;28(13):2473-2481. doi: https://doi.org/10.1016/j.vaccine.2010.01.047

Ellis-Iversen J, Cook AJC, Watson E, Nielen M, Larkin L, Wooldridge M, et al. Perceptions, circumstances and motivators that influence implementation of zoonotic control programs on cattle farms. Prev Vet Med. 2010;93(4):276–85. doi: 10.1016/j.prevetmed.2009.11.005

Enticott G, Maye D, Carmody P, Naylor R, Ward K, Hinchliffe S, et al. Farming on the edge: Farmer attitudes to bovine tuberculosis in newly endemic areas. Vet Rec. 2015;177(17):439. doi: 10.1136/vr.103187

Espetvedt MN, Rintakoski S, Wolff C, Lind AK, Lindberg A, Virtala AMK. Nordic veterinarians’ threshold for medical treatment of dairy cows, influence on disease recording and medicine use: Mild clinical mastitis as an example. Prev Vet Med. 2013;112(1–2):76–89. 10.1016/j.prevetmed.2013.07.004

Espetvedt M, Lind AK, Wolff C, Rintakoski S, Virtala AM, Lindberg A. Nordic dairy farmers’ threshold for contacting a veterinarian and consequences for disease recording: Mild clinical mastitis as an example. Prev Vet Med. 2013;108(2–3):114–24. doi: 10.1016/j.prevetmed.2012.07.014

Fandamu P, Thys E, Duchateau L, Berkvens D. Perception of cattle farmers of the efficacy of east coast fever immunization in Southern Zambia. Trop Anim Health Prod. 2006;38(1):9–16. doi: 10.1007/s11250-006-4341-4

Fischer K, Sjöström K, Stiernström A, Emanuelson U. Dairy farmers’ perspectives on antibiotic use: A qualitative study. J Dairy Sci. 2019;102(3):2724–37. doi: 10.3168/jds.2018-15015

Freitas LN, Cerqueira PHR, Marques HZ, Leandro RA, Machado PF. Human behavioral influences and milk quality control programs. Animal. 2018;12(3):606–11. doi: 10.1017/S1751731117001756

Friedman DB, Kanwat CP, Headrick ML, Patterson NJ, Neely JC, Smith LU. Importance of prudent antibiotic use on dairy farms in South Carolina: A pilot project on farmers’ knowledge, attitudes and practices. Zoonoses Public Health. 2007;54(9–10):366–75. doi: 10.1111/j.1863-2378.2007.01077.x

Frössling J, Nöremark M. Differing perceptions – Swedish farmers’ views of infectious disease control. Vet Med Sci. 2016;2(1):54–68. doi: 10.1002/vms3.20

Gates MC, Evans CA, Han JH, Heuer C, Weston JF. Practices and opinions of New Zealand beef cattle farmers towards bovine viral diarrhoea control in relation to real and perceived herd serological status. N Z Vet J. 2020;68(2):92–100. doi: 10.1080/00480169.2019.1692735

Gerber M, Dürr S, Bodmer M. Decision-making of Swiss farmers and the role of the veterinarian in reducing antimicrobial use on dairy farms. Front Vet Sci. 2020;7:565. doi: 10.3389/fvets.2020.00565

Gethmann J, Zilow V, Probst C, Elbers ARW, Conraths FJ. Why German farmers have their animals vaccinated against Bluetongue virus serotype 8: Results of a questionnaire survey. Vaccine. 2015;33(1):214–21. doi: 10.1016/j.vaccine.2014.10.025

Golding SE, Ogden J, Higgins HM. Shared goals, different barriers: A qualitative study of UK veterinarians’ and farmers’ beliefs about antimicrobial resistance and stewardship. Front Vet Sci. 2019;6(APR):132. doi: 10.3389/fvets.2019.00132

Gunn GJ, Heffernan C, Hall M, McLeod A, Hovi M. Measuring and comparing constraints to improved biosecurity amongst GB farmers, veterinarians and the auxiliary industries. Prev Vet Med. 2008;84(3–4):310–23. doi: 10.1016/j.prevetmed.2007.12.003

Hall J, Wapenaar W. Opinions and practices of veterinarians and dairy farmers towards herd health management in the UK. Vet Rec. 2012;170(17):441. doi: 10.1136/vr.100318

Hamilton L, Evans N, Allcock J. “I don’t go to meetings”: Understanding farmer perspectives on bovine TB and biosecurity training. Vet Rec. 2019;184(13):410. doi: 10.1136/vr.104995

Hansson H, Lagerkvist CJ. Decision making for animal health and welfare: Integrating risk-benefit analysis with prospect theory. Risk Anal. 2014;34(6):1149–59. doi: 10.1111/risa.12154

Hardefeldt LY, Gilkerson JR, Billman‐Jacobe H, Stevenson MA, Thursky K, Bailey KE & Browning GF. Barriers to and enablers of implementing antimicrobial stewardship programs in veterinary practices. J. Vet. Intern. Med. 2018;32(3): 1092-1099. doi: https://doi.org/10.1111/jvim.15083

Heffernan C, Azbel-Jackson L, Brownlie J, Gunn G. Farmer attitudes and livestock disease: Exploring citizenship behaviour and peer monitoring across two BVD control schemes in the UK. PLoS One. 2016;11(3):e0152295. doi: 10.1371/journal.pone.0152295

Heffernan C, Nielsen L, Thomson K, Gunn G. An exploration of the drivers to bio-security collective action among a sample of UK cattle and sheep farmers. Prev Vet Med. 2008;87(3–4):358–72. doi: 10.1016/j.prevetmed.2008.05.007

Hektoen L. Investigations of the motivation underlying Norwegian dairy farmers’ use of homoeopathy. Vet Rec. 2004;155(22):701–7. doi: 10.1136/vr.155.22.701

Helliwell R, Morris C, Raman S. Can resistant infections be perceptible in UK dairy farming? Palgrave Commun. 2019;5:12. doi: 10.1057/s41599-019-0220-2

Hidano A, Gates MC, Enticott G. Farmers’ decision making on livestock trading practices: Cowshed culture and behavioral triggers amongst New Zealand dairy farmers. Front Vet Sci. 2019;6:320. doi: 10.3389/fvets.2019.00320

Higgins HM, Golding SE, Mouncey J, Nanjiani I, Cook AJC. Understanding veterinarians’ prescribing decisions on antibiotic dry cow therapy. J Dairy Sci. 2017;100(4):2909–16. doi: 10.3168/jds.2016-11923

Higgins V, Bryant M, Hernandez-Jover M, Rast L, McShane C. Devolved responsibility and on-farm biosecurity: Practices of biosecure farming care in livestock production. Sociol Ruralis. 2018 Jan;58(1):20–39. doi: https://doi.org/10.1111/soru.12155

Jansen J, Steuten CDM, Renes RJ, Aarts N, Lam TJGM. Debunking the myth of the hard-to-reach farmer: Effective communication on udder health. J Dairy Sci. 2010;93(3):1296–306. doi: 10.3168/jds.2009-2794

Jansen J, van den Borne BHP, Renes RJ, van Schaik G, Lam TJGM, Leeuwis C. Explaining mastitis incidence in Dutch dairy farming: The influence of farmers’ attitudes and behaviour. Prev Vet Med. 2009;92(3):210–23. doi: 10.1016/j.prevetmed.2009.08.015

Jansen J, van Schaik G, Renes RJ, Lam TJGM. The effect of a national mastitis control program on the attitudes, knowledge, and behavior of farmers in the Netherlands. J Dairy Sci. 2010;93(12):5737–47. doi: 10.3168/jds.2010-3318

Jansen J, Lam TJGM. The role of communication in improving udder health. Vet Clin North Am - Food Anim Pract. 2012;28(2):363–79. doi: 10.1016/j.cvfa.2012.03.003

Jemberu WT, Molla W, Dagnew T, Rushton J, Hogeveen H. Farmers’ willingness to pay for foot and mouth disease vaccine in different cattle production systems in Amhara region of Ethiopia. PLoS One. 2020;15:e0239829. doi: 10.1371/journal.pone.0239829

Jemberu WT, Mourits MCM, Hogeveen H. Farmers’ intentions to implement foot and mouth disease control measures in Ethiopia. PLoS One. 2015;10(9):e0138363. doi: 10.1371/journal.pone.0138363

Jones PJ, Sok J, Tranter RB, Blanco-Penedo I, Fall N, Fourichon C, et al. Assessing, and understanding, European organic dairy farmers’ intentions to improve herd health. Prev Vet Med. 2016;133:84–96. doi: 10.1016/j.prevetmed.2016.08.005

Jones PJ, Marier EA, Tranter RB, Wu G, Watson E & Teale CJ. Factors affecting dairy farmers’ attitudes towards antimicrobial medicine usage in cattle in England and Wales. Prev Vet Med. 2015;121(1-2): 30-40. doi: 10.1016/j.prevetmed.2015.05.010

Jonsson NN, Matschoss AL. Attitudes and practices of Queensland dairy farmers to the control of the cattle tick, *Boophilus microplus*. Aust Vet J. 1998;76(11):746–51. doi: 10.1111/j.1751-0813.1998.tb12306.x

Kramer T, Jansen LE, Lipman LJA, Smit LAM, Heederik DJJ, Dorado-García A. Farmers’ knowledge and expectations of antimicrobial use and resistance are strongly related to usage in Dutch livestock sectors. Prev Vet Med. 2017;147:142–8. doi: 10.1016/j.prevetmed.2017.08.023

Kristensen E, Enevoldsen C. A mixed methods inquiry: How dairy farmers perceive the value(s) of their involvement in an intensive dairy herd health management program. Acta Vet Scand. 2008;50(1):50. doi: 10.1186/1751-0147-50-50

Kristensen E, Jakobsen EB. Danish dairy farmers’ perception of biosecurity. Prev Vet Med. 2011;99(2–4):122–9. doi: 10.1016/j.prevetmed.2011.01.010

Kumar V, Gupta J, Meena HR. Assessment of awareness about antibiotic resistance and practices followed by veterinarians for judicious prescription of antibiotics: An exploratory study in Eastern Haryana region of India. Trop Anim Health Prod. 2019;51(3):677–87. doi: 10.1007/s11250-018-1742-0

Kuster K, Cousin ME, Jemmi T, Schöpbach-Regula G, Magouras I. Expert opinion on the perceived effectiveness and importance of on-farm biosecurity measures for cattle and swine farms in Switzerland. PLoS One. 2015;10(12):e0144533. doi: 10.1371/journal.pone.0144533

Laanen M, Maes D, Hendriksen C, Gelaude P, De Vliegher S, Rosseel Y, et al. Pig, cattle and poultry farmers with a known interest in research have comparable perspectives on disease prevention and on-farm biosecurity. Prev Vet Med. 2014;115(1–2):1–9. doi: 10.1016/j.prevetmed.2014.03.015

Lahuerta-Marin A, Brennan ML, Finney G, O’Hagan MJH, Jack C. Key actors in driving behavioural change in relation to on-farm biosecurity; A Northern Ireland perspective. Ir Vet J. 2018;71(1):14. doi: 10.1186/s13620-018-0125-1

Lam TJGM, Van Den Borne BHP, Jansen J, Huijps K, Van Veersen JCL, Van Schaik G, et al. Improving bovine udder health: A national mastitis control program in the Netherlands. J Dairy Sci. 2013;96(2):1301–11. doi: 10.3168/jds.2012-5958

Lanyon SR, Anderson ML, Reichel MP. Survey of farmer knowledge and attitudes to endemic disease management in South Australia, with a focus on bovine viral diarrhoea (bovine pestivirus). Aust Vet J. 2015;93(5):157–63. doi: 10.1111/avj.12316

Leach KA, Paul ES, Whay HR, Barker ZE, Maggs CM, Sedgwick AK, et al. Reducing lameness in dairy herds - Overcoming some barriers. Res Vet Sci. 2013;94(3):820–5. doi: 10.1016/j.rvsc.2012.10.005

Leach KA, Whay HR, Maggs CM, Barker ZE, Paul ES, Bell AK, et al. Working towards a reduction in cattle lameness: 1. Understanding barriers to lameness control on dairy farms. Res Vet Sci. 2010;89(2):311–7. doi: 10.1016/j.rvsc.2010.02.014

Leach KA, Whay HR, Maggs CM, Barker ZE, Paul ES, Bell AK, et al. Working towards a reduction in cattle lameness: 2. Understanding dairy farmers’ motivations. Res Vet Sci. 2010;89(2):318–23. doi: 10.1016/j.rvsc.2010.02.017

Lestari VS, Rahardja DP, Mappigau P, Rohani ST, Sirajuddin SN. Beef cattle farmers behavior toward biosecurity. J Indones Trop Anim Agric. 2019;44(2):204–12.

Lestari VS, Sirajuddin SN & Abdullah A. Constraints of biosecurity adoption on beef cattle farms. Eur J Sustain Dev. 2018;7(3):151-151. doi: 10.14207/ejsd.2018.v7n3p151

Lind AK, Thomsen PT, Rintakoski S, Espetvedt MN, Wolff C, Houe H. The association between farmers’ participation in herd health programmes and their behaviour concerning treatment of mild clinical mastitis. Acta Vet Scand. 2012;54:62. doi: 10.1186/1751-0147-54-62

Lind N, Hansson H, Emanuelson U, Lagerkvist CJ. A combination of differentiation and consolidation theory and risk-benefit analysis to examine decisions on mastitis prevention. J Risk Res. 2020;23(2):194–209. doi: 10.1080/13669877.2018.1547783

Little R, Wheeler K, Edge S. Developing a risk-based trading scheme for cattle in England: Farmer perspectives on managing trading risk for bovine tuberculosis. Vet Rec. 2017;180(6):148. doi: 10.1136/vr.103522

Mahon MM, Sheehan MC, Kelleher PF, Johnson AJ, Doyle SM. An assessment of Irish farmers’ knowledge of the risk of spread of infection from animals to humans and their transmission prevention practices. Epidemiol Infect. 2017;145(12):2424–35. doi: 10.1017/S0950268817001418

Makita K, Steenbergen E, Haruta L, Hossain S, Nakahara Y, Tamura Y, et al. Quantitative understanding of the decision-making process for farm biosecurity among Japanese livestock farmers using the KAP-capacity framework. Front Vet Sci. 2020;7:614. doi: 10.3389/fvets.2020.00614

Maye D, Enticott G, Naylor R. Theories of change in rural policy evaluation. Sociol Ruralis. 2020 Jan;60(1):198–221. doi: 10.1111/soru.12269

Maye D, Enticott G & Naylor R. Using scenario-based influence mapping to examine farmers’ biosecurity behaviour. Land use policy. 2017;66:265-277. doi: https://doi.org/10.1016/j.landusepol.2017.04.026

McAloon CG, Macken-Walsh Á, Moran L, Whyte P, More SJ, O’Grady L, et al. Johne’s disease in the eyes of Irish cattle farmers: A qualitative narrative research approach to understanding implications for disease management. Prev Vet Med. 2017;141:7–13. doi: 10.1016/j.prevetmed.2017.04.001

McFarland L, Macken-Walsh Á, Claydon G, Casey M, Douglass A, McGrath G, et al. Irish dairy farmers’ engagement with animal health surveillance services: Factors influencing sample submission. J Dairy Sci. 2020;103(11):10614–27. doi: 10.3168/jds.2019-17889

McLeod E, Jensen K, Griffith AP, Delong KL. Tennessee beef producers’ willingness to participate in a state-branded beef program. J Agric Appl Econ. 2018 Nov;50(4):579–601. doi: DOI: 10.1017/aae.2018.16

Mekonnen SA, Koop G, Lam TJGM, Hogeveen H. The intention of North-Western Ethiopian dairy farmers to control mastitis. PLoS One. 2017;12(8):e0182727. doi: 10.1371/journal.pone.0182727

Mills KE, Weary DM, von Keyserlingk MAG. Identifying barriers to successful dairy cow transition management. J Dairy Sci. 2020;103(2):1749–58. doi: 10.3168/jds.2018-16231

Mingolla C, Hudders L, Vanwesenbeeck I, Claerebout E. Towards a biased mindset: An extended Theory of Planned Behaviour framework to predict farmers’ intention to adopt a sustainable mange control approach. Prev Vet Med. 2019;169:104695. doi: 10.1016/j.prevetmed.2019.104695

Moya S, Tirado F, Espluga J, Ciaravino G, Armengol R, Diéguez J, et al. Dairy farmers’ decision-making to implement biosecurity measures: A study of psychosocial factors. Transbound Emerg Dis. 2020;67(2):698–710. doi: 10.1111/tbed.13387

Naylor R, Courtney P. Exploring the social context of risk perception and behaviour: Farmers’ response to bovine tuberculosis. Geoforum. 2014;57:48–56. doi: https://doi.org/10.1016/j.geoforum.2014.08.011

Nöremark M, Sternberg-Lewerin S. On-farm biosecurity as perceived by professionals visiting Swedish farms. Acta Vet Scand. 2014;56:28. doi: 10.1186/1751-0147-56-28

Nöremark M, Sternberg Lewerin S, Ernholm L & Frössling J. Swedish farmers’ opinions about biosecurity and their intention to make professionals use clean protective clothing when entering the stable. Front Vet Sci. 2016;3:46. doi: 10.3389/fvets.2016.00046

O’Hagan MJH, Matthews DI, Laird C, McDowell SWJ. Farmers’ beliefs about bovine tuberculosis control in Northern Ireland. Vet J. 2016;212:22–6. doi: 10.1016/j.tvjl.2015.10.038

Ochieng BJ, Hobbs JE. Factors affecting cattle producers’ willingness to adopt an *Escherichia coli* O157:H7 vaccine: a probit analysis. Int Food Agribusiness Manag Rev. 2016;20:347–63. doi: 10.22434/IFAMR2016.0177

Poizat A, Bonnet-Beaugrand F, Rault A, Fourichon C, Bareille N. Antibiotic use by farmers to control mastitis as influenced by health advice and dairy farming systems. Prev Vet Med. 2017;146:61–72. doi: 10.1016/j.prevetmed.2017.07.016

Pozza G, Pinto A, Crovato S, Mascarello G, Bano L, Dacasto M, Marangon S, et al. Antimicrobial use and antimicrobial resistance: standpoint and prescribing behaviour of Italian cattle and pig veterinarians. Ital J Anim Sci. 2020;19(1); 905-916. doi: 10.1080/1828051X.2020.1807419

Pucken VB, Schüpbach-Regula G, Gerber M, Gross CS, Bodmer M. Veterinary peer study groups as a method of continuous education—A new approach to identify and address factors associated with antimicrobial prescribing. PLoS One. 2019;14(9):e0222497. doi: 10.1371/journal.pone.0222497

Rathod P, Chander M, Bangar Y. Livestock vaccination in India: an analysis of theory and practice among multiple stakeholders. Rev Sci Tech Off Int Epiz. 2016;35(3): 729-39.

Redding LE, Barg FK, Smith G, Galligan DT, Levy MZ & Hennessy S. The role of veterinarians and feed-store vendors in the prescription and use of antibiotics on small dairy farms in rural Peru. J Dairy Sci. 2013;96(11): 7349-7354. doi: 10.3168/jds.2013-7045

Rell J, Wunsch N, Home R, Kaske M, Walkenhorst M, Vaarst M. Stakeholders’ perceptions of the challenges to improving calf health and reducing antimicrobial use in Swiss veal production. Prev Vet Med. 2020;179:104970. doi: 10.1016/j.prevetmed.2020.104970

Relun A, Guatteo R, Auzanneau MM, Bareille N. Farmers’ practices, motivators and barriers for adoption of treatments of digital dermatitis in dairy farms. Animal. 2013;7(9):1542–50. doi: 10.1017/S1751731113000803

Renault V, Damiaans B, Sarrazin S, Humblet MF, Dewulf J, Saegerman C. Biosecurity practices in Belgian cattle farming: Level of implementation, constraints and weaknesses. Transbound Emerg Dis. 2018;65(5):1246–61. doi: 10.1111/tbed.12865

Renault V, Humblet MF, Moons V, Bosquet G, Gauthier B, Cebrián LM, et al. Rural veterinarian’s perception and practices in terms of biosecurity across three European countries. Transbound Emerg Dis. 2018;65(1):e183–93. doi: 10.1111/tbed.12719

Renault V, Damiaans B, Humblet MF, Jiménez Ruiz S, García Bocanegra I, Brennan ML, et
 al. Cattle farmers’ perception of biosecurity measures and the main predictors of
 behaviour change: The first European-wide pilot study. Transbound Emerg Dis. 2020;
 in press. doi: [10.1111/tbed.13935](https://doi.org/10.1111/tbed.13935)

Richens IF, Hobson-West P, Brennan ML, Hood Z, Kaler J, Green M, et al. Factors influencing veterinary surgeons’ decision-making about dairy cattle vaccination. Vet Rec. 2016;179(16):410. doi: 10.1136/vr.103822

Richens IF, Hobson-West P, Brennan ML, Lowton R, Kaler J, Wapenaar W. Farmers’ perception of the role of veterinary surgeons in vaccination strategies on British dairy farms. Vet Rec. 2015;177(18):465. doi: 10.1136/vr.103415

Richens IF, Houdmont J, Wapenaar W, Shortall O, Kaler J, O’Connor H, et al. Application of multiple behaviour change models to identify determinants of farmers’ biosecurity attitudes and behaviours. Prev Vet Med. 2018;155:61–74. doi: 10.1016/j.prevetmed.2018.04.010

Rink KA, Turk P, Archibeque-Engle SL, Wilmer H, Ahola JK, Hadrich JC, et al. Dairy producer perceptions of the Farmers Assuring Responsible Management (FARM) Animal Care Program. J Dairy Sci. 2019;102(12):11317–27. doi: 10.3168/jds.2019-16859

Ritter C, Kwong GPS, Wolf R, Pickel C, Slomp M, Flaig J, et al. Factors associated with participation of Alberta dairy farmers in a voluntary, management-based Johne’s disease control program. J Dairy Sci. 2015;98(11):7831–45. 10.3168/jds.2015-9789

Ritter C, Jansen J, Roth K, Kastelic JP, Adams CL, Barkema HW. Dairy farmers’ perceptions toward the implementation of on-farm Johne’s disease prevention and control strategies. J Dairy Sci. 2016;99(11):9114–25. doi: 10.3168/jds.2016-10896

Ritter C, Adams CL, Kelton DF, Barkema HW. Factors associated with dairy farmers’ satisfaction and preparedness to adopt recommendations after veterinary herd health visits. J Dairy Sci. 2019;102(5):4280–93. doi: 10.3168/jds.2018-15825

Ritter C, Mills KE, Weary DM, von Keyserlingk MAG. Perspectives of western Canadian dairy farmers on the future of farming. J Dairy Sci. 2020;103(11):10273–82. doi: 10.3168/jds.2020-18430

Robinson PA. “They’ve got to be testing and doing something about it”: Farmer and veterinarian views on drivers for Johne’s disease control in dairy herds in England. Prev Vet Med. 2020;182:105094. doi: 10.1016/j.prevetmed.2020.105094

Roche SM, Jones-Bitton A, Meehan M, Von Massow M, Kelton DF. Evaluating the effect of focus farms on Ontario dairy producers’ knowledge, attitudes, and behavior toward control of Johne’s disease. J Dairy Sci. 2015;98(8):5222–40. doi: 10.3168/jds.2014-8765

Roche SM, Kelton DF, Meehan M, Von Massow M, Jones-Bitton A. Exploring dairy producer and veterinarian perceptions of barriers and motivators to adopting on-farm management practices for Johne’s disease control in Ontario, Canada. J Dairy Sci. 2019;102(5):4476–88. doi: 10.3168/jds.2018-15944

Santman-Berends IMGA, Buddiger M, Smolenaars AJG, Steuten CDM, Roos CAJ, Van Erp AJM, et al. A multidisciplinary approach to determine factors associated with calf rearing practices and calf mortality in dairy herds. Prev Vet Med. 2014;117(2):375–87. doi: 10.1016/j.prevetmed.2014.07.011

Sawford K, Vollman AR, Stephen C. A focused ethnographic study of Alberta cattle veterinarians’ decision making about diagnostic laboratory submissions and perceptions of surveillance programs. PLoS One. 2013;8(5):e64811. doi: 10.1371/journal.pone.0064811

Sayers RG, Sayers GP, Mee JF, Good M, Bermingham ML, Grant J, et al. Implementing biosecurity measures on dairy farms in Ireland. Vet J. 2013;197(2):259–67. doi: 10.1016/j.tvjl.2012.11.017

Sayers RG, Good M, Sayers GP. A survey of biosecurity-related practices, opinions and communications across dairy farm veterinarians and advisors. Vet J. 2014;200(2):261–9. doi: 10.1016/j.tvjl.2014.02.010

Scherpenzeel CGM, Santman-Berends IMGA, Lam TJGM. Veterinarians’ attitudes toward antimicrobial use and selective dry cow treatment in the Netherlands. J Dairy Sci. 2018;101(7):6336–45. doi: 10.3168/jds.2017-13591

Scherpenzeel CGM, Tijs SHW, den Uijl IEM, Santman-Berends IMGA, Velthuis AGJ, Lam TJGM. Farmers’ attitude toward the introduction of selective dry cow therapy. J Dairy Sci. 2016;99(10):8259–66. doi: 10.3168/jds.2016-11349

Schwendner AA, Lam TJGM, Bodmer M, Cousin ME, Schüpbach-Regula G, van den Borne BHP. Knowledge, attitude and practices of Swiss dairy farmers towards intramammary antimicrobial use and antimicrobial resistance: A latent class analysis. Prev Vet Med. 2020;179:105023. doi: 10.1016/j.prevetmed.2020.105023

Sharma G, Mutua F, Deka RP, Shome R, Bandyopadhyay S, Shome BR, et al. A qualitative study on antibiotic use and animal health management in smallholder dairy farms of four regions of India. Infect Ecol Epidemiol. 2020;10(1):1792033. doi: 10.1080/20008686.2020.1792033

Shock DA, Coe JB, LeBlanc SJ, Leslie KE, Renaud D, Roche S, et al. Characterizing the attitudes and motivations of Ontario dairy producers toward udder health. J Dairy Sci. 2020;103(5):4618–32. doi: 10.3168/jds.2019-16621

Shortall O, Ruston A, Green M, Brennan M, Wapenaar W, Kaler J. Broken biosecurity? Veterinarians’ framing of biosecurity on dairy farms in England. Prev Vet Med. 2016;132:20–31. doi: 10.1016/j.prevetmed.2016.06.001

Shortall O, Sutherland L, Ruston A, Kaler J. True cowmen and commercial farmers: exploring vets’ and dairy farmers’ contrasting views of ‘good farming’ in relation to biosecurity. Sociol Ruralis. 2018;58(3):583–603.

Sok J, Hogeveen H, Elbers ARW, Lansink AGJMO. Perceived risk and personality traits explaining heterogeneity in Dutch dairy farmers’ beliefs about vaccination against Bluetongue. J Risk Res. 2018;21(5):562–78. doi: 10.1080/13669877.2016.1223162

Sok J, Hogeveen H, Elbers ARW, Oude Lansink AGJM. Using farmers’ attitude and social pressures to design voluntary Bluetongue vaccination strategies. Prev Vet Med. 2016;133:114–9. doi: 10.1016/j.prevetmed.2016.09.016

Sorge US, Mount J, Kelton DF, Godkin A. Veterinarians’ perspective on a voluntary Johne’s disease prevention program in Ontario and western Canada. Can Vet J. 2010;51(4):403–5.

Sorge U, Kelton D, Lissemore K, Godkin A, Hendrick S & Wells S. Attitudes of Canadian dairy farmers toward a voluntary Johne's disease control program. J Dairy Sci. 2010;93(4);1491-1499. doi: 10.3168/jds.2009-2447

Speksnijder DC, Jaarsma ADC, van der Gugten AC, Verheij TJM, Wagenaar JA. Determinants associated with veterinary antimicrobial prescribing in farm animals in the Netherlands: a qualitative study. Zoonoses Public Health. 2015;62 Suppl 1:39–51. doi: 10.1111/zph.12168

Suit-B Y, Hassan L, Krauss SE, Ramanoon SZ, Ooi PT, Yasmin AR, et al. Exploring the mental model of cattle farmers in disease prevention and control practices. Vet Sci. 2020;7(1):27. doi: 10.3390/vetsci7010027

Suleiman A, Jackson E, Rushton J. Perceptions, circumstances and motivators affecting the implementation of contagious bovine pleuropneumonia control programmes in Nigerian Fulani pastoral herds. Prev Vet Med. 2018;149:67–74. doi: 10.1016/j.prevetmed.2017.10.011

Sungirai M, Moyo DZ, De Clercq P, Madder M. Communal farmers’ perceptions of tick-borne diseases affecting cattle and investigation of tick control methods practiced in Zimbabwe. Ticks Tick Borne Dis. 2016;7(1):1–9. doi: 10.1016/j.ttbdis.2015.07.015

Svensson C, Alvåsen K, Eldh AC, Frössling J, Lomander H. Veterinary herd health management–Experience among farmers and farm managers in Swedish dairy production. Prev Vet Med. 2018;155:45–52. doi: 10.1016/j.prevetmed.2018.04.012

Svensson C, Lind N, Reyher KK, Bard AM, Emanuelson U. Trust, feasibility, and priorities influence Swedish dairy farmers’ adherence and nonadherence to veterinary advice. J Dairy Sci. 2019;102(11):10360–8. doi: 10.3168/jds.2019-16470

Swinkels JM, Hilkens A, Zoche-Golob V, Krömker V, Buddiger M, Jansen J, et al. Social influences on the duration of antibiotic treatment of clinical mastitis in dairy cows. J Dairy Sci. 2015;98(4):2369–80. doi: 10.3168/jds.2014-8488

Taylor DD, Martin JN, Morley PS, Belk KE, White AE, Walter EJS. Survey of production animal veterinarians’ prescription practices, factors influencing antimicrobial drug use, and perceptions of and attitudes toward antimicrobial resistance. J Am Vet Med Assoc. 2020;257(1):87–96. doi: 10.2460/JAVMA.257.1.87

Toma L, Low JC, Vosough Ahmadi B, Matthews L, Stott AW. An analysis of cattle farmers’ perceptions of drivers and barriers to on-farm control of Escherichia coli O157. Epidemiol Infect. 2015;143(11):2355–66. doi: 10.1017/S0950268814003045

Toma L, Stott AW, Heffernan C, Ringrose S, Gunn GJ. Determinants of biosecurity behaviour of British cattle and sheep farmers-A behavioural economics analysis. Prev Vet Med. 2013;108(4):321–33. doi: 10.1016/j.prevetmed.2012.11.009

Truong DB, Binot A, Peyre M, Nguyen NH, Bertagnoli S, Goutard FL. A Q method approach to evaluating farmers’ perceptions of foot-and-mouth disease vaccination in Vietnam. Front Vet Sci. 2017;4(JUN):95. doi: 10.3389/fvets.2017.00095

Tunstall J, Mueller K, White DG, Oultram JWH, Higgins HM. Lameness in beef cattle: UK farmers’ perceptions, knowledge, barriers, and approaches to treatment and control. Front Vet Sci. 2019;6(MAR):94. doi: 10.3389/fvets.2019.00094

Vaarst M, Sørensen JT. Danish dairy farmers’ perceptions and attitudes related to calf-management in situations of high versus no calf mortality. Prev Vet Med. 2009;89(1–2):128–33. doi: 10.1016/j.prevetmed.2009.02.015

Vaarst M, Paarup-Laursen B, Houe H, Fossing C & Andersen HJ. Farmers’ choice of medical treatment of mastitis in Danish dairy herds based on qualitative research interviews. J Dairy Sci. 2002;85(4):992-1001.

Valeeva NI, Lam TJGM, Hogeveen H. Motivation of dairy farmers to improve mastitis management. J Dairy Sci. 2007;90(9):4466–77. doi: 10.3168/jds.2007-0095

Vande Velde F, Charlier J, Hudders L, Cauberghe V, Claerebout E. Beliefs, intentions, and beyond: A qualitative study on the adoption of sustainable gastrointestinal nematode control practices in Flanders’ dairy industry. Prev Vet Med. 2018;153:15–23. doi: 10.1016/j.prevetmed.2018.02.020

Vande Velde F, Claerebout E, Cauberghe V, Hudders L, Van Loo H, Vercruysse J, et al. Diagnosis before treatment: Identifying dairy farmers’ determinants for the adoption of sustainable practices in gastrointestinal nematode control. Vet Parasitol. 2015;212(3–4):308–17. doi: 10.1016/j.vetpar.2015.07.013

Vasquez AK, Foditsch C, Dulièpre SAC, Siler JD, Just DR, Warnick LD, et al. Understanding the effect of producers’ attitudes, perceived norms, and perceived behavioral control on intentions to use antimicrobials prudently on New York dairy farms. PLoS One. 2019;14(9):e0222442. doi: 10.1371/journal.pone.0222442

Vissio C, Richardet M, Chaves J & Larriestra A. Preference of veterinarians to select an udder health programme for milk producers. Vet Rec Open. 2019;6(1):e000313. doi:10.1136/ vetreco-2018-000313

Warren M, Lobley M, Winter M. Farmer attitudes to vaccination and culling of badgers in controlling bovine tuberculosis. Vet Rec. 2013;173(2):40. doi: 10.1136/vr.101601

Wemette M, Safi AG, Beauvais W, Ceres K, Shapiro M, Moroni P, et al. New York State dairy farmers’ perceptions of antibiotic use and resistance: A qualitative interview study. PLoS One. 2020;15(5):e0232937. doi: 10.1371/journal.pone.0232937

Wiley KE, Walker J, Lower T, Massey PD, Durrheim DN, Khandaker G. Australian beef industry worker’s knowledge, attitudes and practices regarding Q fever: A pilot study. Vaccine. 2019;37(43):6336–41. doi: 10.1016/j.vaccine.2019.09.020

Wilson CS, Jenkins DJ, Barnes TS, Brookes VJ. Australian beef producers’ knowledge and attitudes relating to hydatid disease are associated with their control practices. Prev Vet Med. 2020;182:105078. doi: 10.1016/j.prevetmed.2020.105078

Wolff C, Abigaba S, Sternberg Lewerin S. Ugandan cattle farmers’ perceived needs of disease prevention and strategies to improve biosecurity. BMC Vet Res. 2019;15(1):208. doi: 10.1186/s12917-019-1961-2
